# WhatsApp Versus SMS for 2-Way, Text-Based Follow-Up After Voluntary Medical Male Circumcision in South Africa: Exploration of Messaging Platform Choice

**DOI:** 10.2196/62762

**Published:** 2024-10-16

**Authors:** Isabella Fabens, Calsile Makhele, Nelson Kibiribiri Igaba, Sizwe Hlongwane, Motshana Phohole, Evelyn Waweru, Femi Oni, Madalitso Khwepeya, Maria Sardini, Khumbulani Moyo, Hannock Tweya, Mourice Barasa Wafula, Jacqueline Pienaar, Felex Ndebele, Geoffrey Setswe, Tracy Qi Dong, Caryl Feldacker

**Affiliations:** 1 International Training and Education Center for Health (I-TECH) Departments of Global Health and Medicine University of Washington Seattle, WA United States; 2 Aurum Institute Johannesburg South Africa; 3 Right to Care Johannesburg South Africa; 4 Medic Nairobi Kenya; 5 Fred Hutchinson Cancer Center Seattle, WA United States

**Keywords:** 2-way texting, text messages, WhatsApp, digital health innovations, male circumcision, South Africa, quality improvement

## Abstract

**Background:**

Telehealth is growing, especially in areas where access to health facilities is difficult. We previously used 2-way texting (2wT) via SMS to improve the quality of postoperative care after voluntary medical male circumcision in South Africa. In this study, we offered males aged 15 years and older WhatsApp or SMS as their message delivery and interaction platform to explore user preferences and behaviors.

**Objective:**

The objectives of this process evaluation embedded within a larger 2wT expansion trial were to (1) explore 2wT client preferences, including client satisfaction, with WhatsApp or SMS; (2) examine response rates (participation) by SMS and WhatsApp; and (3) gather feedback from the 2wT implementation team on the WhatsApp approach.

**Methods:**

Males aged 15 years and older undergoing voluntary medical male circumcision in program sites could choose their follow-up approach, selecting 2wT via SMS or WhatsApp or routine care (in-person postoperative visits). The 2wT system provided 1-way educational messages and an open 2-way communication channel between providers and clients. We analyzed quantitative data from the 2wT database on message delivery platforms (WhatsApp vs SMS), response rates, and user behaviors using chi-square tests, *z* tests, and *t* tests. The team conducted short phone calls with WhatsApp and SMS clients about their perceptions of this 2wT platform using a short, structured interview guide. We consider informal reflections from the technical team members on the use of WhatsApp. We applied an implementation science lens using the RE-AIM (reach, effectiveness, adoption, implementation, and maintenance) framework to focus results on practice and policy improvement.

**Results:**

Over a 2-month period—from August to October, 2023—337 males enrolled in 2wT and were offered WhatsApp or SMS and were included in the analysis. For 2wT reach, 177 (53%) participants chose WhatsApp as their platform (*P*=.38). Mean client age was 30 years, and 253 (75%) participants chose English for automated messages. From quality assurance calls, almost all respondents (87/89, 98%) were happy with the way they were followed up. For effectiveness, on average for the days on which responses were requested, 58 (33%) WhatsApp clients and 44 (28%) SMS clients responded (*P*=.50). All 2wT team members believed WhatsApp limited the automated message content, language choices, and inclusivity as compared with the SMS-based 2wT approach.

**Conclusions:**

When presented with a choice of 2wT communication platform, clients appear evenly split between SMS and WhatsApp. However, WhatsApp requires a smartphone and data plan, potentially reducing reach at scale. Clients using both platforms responded to 2wT interactive prompts, demonstrating similar effectiveness in engaging clients in follow-up. For telehealth interventions, digital health designers should maintain an SMS-based platform and carefully consider adding WhatsApp as an option for clients, using an implementation science approach to present evidence that guides the best implementation approach for their setting.

## Introduction

Telehealth, the process of engaging clients and their clinicians via internet, phone, or other digital interfaces, is increasingly recognized for its potential ability to enhance the quality of patient care, including for postoperative follow-up [1-3]. It is also being recognized by guideline-setting organizations: the World Health Organization’s (WHO) Global Strategy on Digital Health 2020-2025 promoted people-centered health systems including the potential contribution of digital, evidence-based self-management tools [4]. However, many digital health interventions aimed at improving follow-up through postoperative education or enhanced communication between providers and patients require the users to have smartphones or web access, resulting in interventions that may be more appropriate in high-resource settings [5-11]. Interventions that require only basic phones or use SMS are less common [12]. Few studies of digital health innovations to improve postoperative care have been conducted in routine sub-Saharan African (SSA) settings or other low- or middle-income country (LMIC) contexts [13-16]. In many LMIC settings, scarce resources, long distances to health care access, and shortages of health care workers (HCWs) reduce easy access to trained clinicians. More evidence on how telehealth can improve the quality of postoperative care in LMIC settings is needed.

Telehealth using mobile phones (mobile health [mHealth]), is an especially helpful tool that reflects the scarcity of HCWs in SSA; as of 2019, there were about 10 times as many nurses and midwives per 1000 people in high-income countries compared with LMICs [17,18]. Mobile phone ownership has been increasing in SSA; 89% of people in SSA have a mobile phone, leading to an increase in the use of mHealth [19]. As only 36% of people in SSA use the internet, SMS is, therefore, a more equitable platform to reach people with only basic, feature phones [20]. As internet access grows, preferences for smartphones, and data-driven communication platforms, like WhatsApp, may become more convenient or desired for their additional functionalities such as photos, videos, and voice messages [21].

Previously, we demonstrated that 2-way texting (2wT) for postoperative follow-up instead of routine, in-person visits was safe, efficient, and acceptable for both clients and providers after voluntary medical male circumcision (VMMC) in South Africa [16,22,23]. A 2wT-based follow-up provides males aged 15 years and older with an option to receive and respond to SMS messages rather than returning for mandatory follow-up visits at the health center on days 2 and 7 after VMMC. Our 2wT approach used SMS and offset use costs for clients, making it free to participate using SMS [24-26]. We found that 2wT identifies complications more quickly than in-person visits [16], saves an average of US $4 per client follow-up [27], and has high usability among clients and their clinicians [22,23].

To explore the use of WhatsApp versus SMS in our routine, South African, postoperative care settings, we embedded a process evaluation within a larger 2wT expansion trial. In this study, our objectives were (1) to explore 2wT client preferences, including client satisfaction, with WhatsApp or SMS; (2) to examine response rates (participation) by SMS and WhatsApp; and (3) to gather feedback from the 2wT implementation team on the WhatsApp approach for 2wT. We apply the lens of the implementation science framework, RE-AIM (reach, effectiveness, adoption, implementation, and maintenance) [28], to better understand individual-level constructs of 2wT reach and effectiveness as well as organization-level implementation using SMS versus WhatsApp. Findings from this evaluation could help inform other mHealth implementers in their consideration of SMS versus WhatsApp as a platform for telehealth delivery in routine LMIC settings.

## Methods

### Background: 2wT Expansion in Routine VMMC Service Delivery

This comparison of WhatsApp to SMS study is nested as the final step within a quasi-experimental, 4-step, stepped wedge study conducted from January 30 to October 19, 2023. The objective of the parent study was to assess the effectiveness and efficiency of 2wT as part of routine VMMC care in Ekurhuleni (Gauteng Province) and Dr Kenneth Kaunda Districts (North West Province). The study was a collaboration between the University of Washington, Seattle, WA USA; Aurum Institute, Johannesburg, South Africa; Right to Care, Pretoria, South Africa; and Medic, Nairobi, Kenya. Eligibility for 2wT included the following: aged≥15 years; possessed a mobile phone during VMMC registration; basic literacy; basic digital literacy; received the 2wT enrollment message; and willing to interact about healing via mobile phone.

In the period from August 17 to October 19, 2023, only, males who opted into the 2wT-based follow-up approach were offered a choice of either SMS or WhatsApp as the platform for the hybrid 2wT message delivery. Nurses who enrolled clients saw the phone the client was using, so only those with smartphones were asked if they wanted to opt into the 2wT approach to receive messages via WhatsApp. Clients were informed that the selection of WhatsApp would require the use of their data plans whereas SMS messages were free to send and receive. This embedded study compares the uptake of 2wT via SMS or WhatsApp among the 2wT intervention group. Forthcoming, broader manuscripts from the complete stepped wedge study will include the entire implementation period, with manuscripts focused on comparing clinical outcomes between 2wT and standard-of-care clients; costing analysis of the 2wT approach in routine settings; and qualitative exploration of barriers and facilitators to 2wT expansion using key informant interviews.

### 2wT Intervention Implementation

**Figure 1:**
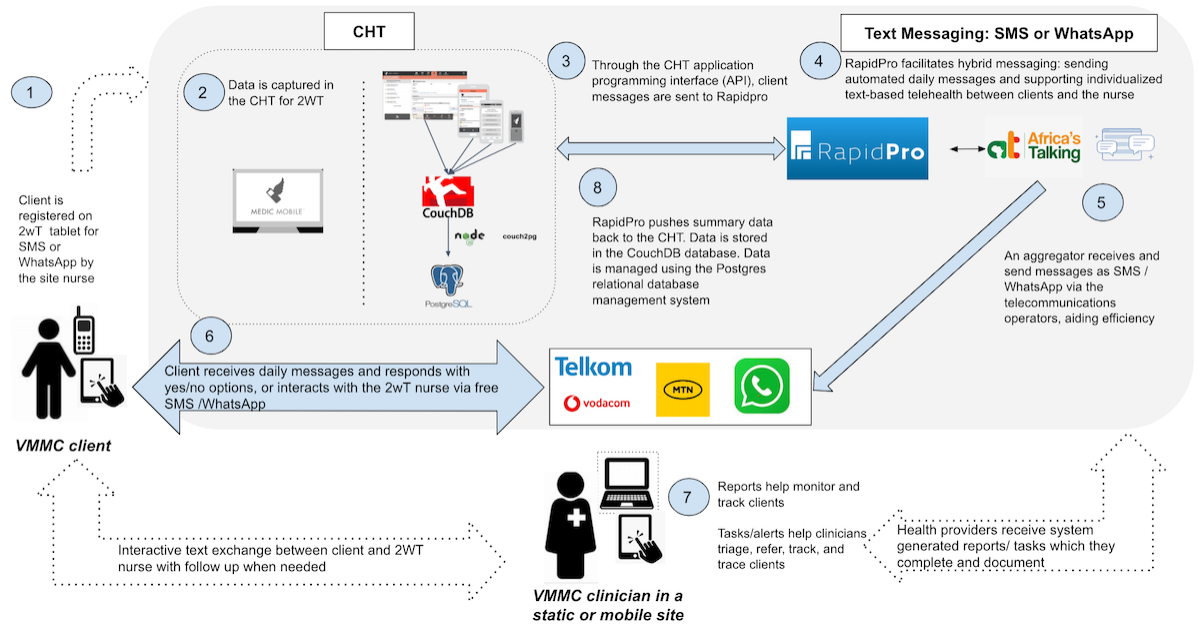
2wT system architecture overview, South Africa (adapted from Feldacker et al [16]) which is published under Creative Commons Attribution 4.0 International License [33]). This figure presents the system architecture for 2wT, both for SMS and WhatsApp. The CHT captures and stores data related to clients. RapidPro and Africa’s Talking enable automated and individualized messages to be sent to and received from, clients through local telecommunications companies. 2wT: 2-way texting; CHT: Community Health Toolkit; VMMC: voluntary medical male circumcision.

The hybrid automated and interactive 2wT intervention, including training details, was described in detail previously [15,16,22,23,29-31]. For the duration of the stepped wedge, there were no major changes to the intervention or open-source software, released previously [32]. The addition of the WhatsApp messaging platform in addition to SMS did not alter the intervention from the perspective of the HCWs; the 2wT interface and message-based interaction remained unchanged (Figure 1). Implementing teams received a brief refresher orientation, including that the updated 2wT enrollment form asked whether participants chose SMS or WhatsApp. Minor downtimes of the WhatsApp server were documented in routine performance monitoring and swiftly addressed.

For 2wT scale-up, clients were made aware of the 2wT follow-up option during VMMC preoperative counseling. Males were offered the choice to be followed up via 2wT or to attend in-person, postoperative visits at the clinic. For those who elected to participate, enrollment into the 2wT system occurred during postoperative counseling. The staff member enrolling the participant physically confirmed that the participant received the confirmation welcome message signaling successful enrollment before discharge.

**Figure 2:**
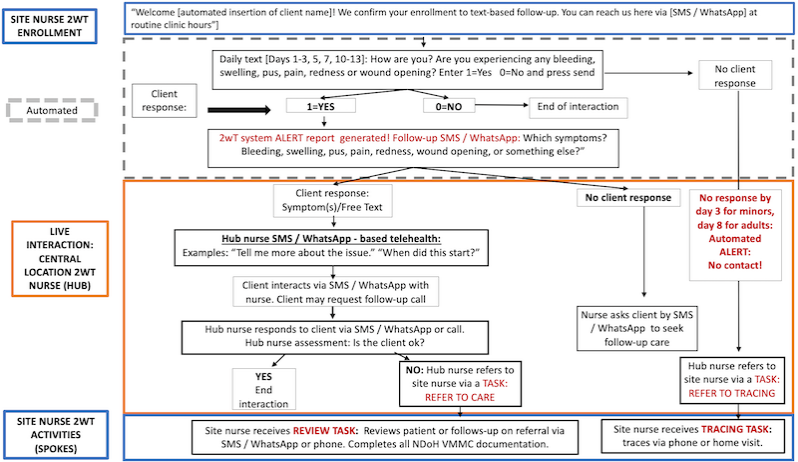
Message flow for the 2wT system (adapted from Feldacker et al [16]) which is published under Creative Commons Attribution 4.0 International License [33]). This figure presents the message flow for the 2wT system. Starting on the day of circumcision, automated prompts ask about healing. Those who respond with potential complications are followed by a “hub nurse,” or a nurse who monitors the entire system. If they need to be seen in person, the hub refers to a nurse at the client’s facility where a “site nurse,” follows up with the client to ensure completion of the referral. The system generates no response “tasks” for health care providers when a client does not respond to any messages, triggering tracing. 2wT: 2-way texting; VMMC: voluntary medical male circumcision.

After enrolling, the 2wT system sent automated messages asking males to respond about their healing progress on days 1-3, 5, 7, 10, and 13 (Figure 2). All 2wT participants were asked to respond with either healing well (responded with “0”), or a complication/request for help (responded with “1”). During the 14-day healing period, the system sent automated educational messages on alternate days about wound care, including how to remove the bandage, properly soak the wound, and elevate it to reduce swelling. Automated messages were the same via WhatsApp or SMS. Clients could respond unprompted to the provider on any day. When males responded with potential complications, a nurse responded via message and referred them for in-person care when necessary. For SMS, males could choose to receive their automated, scripted messages in Afrikaans, English, Sotho, Tswana, or Zulu. Those who chose WhatsApp could only choose between 3 of the languages, Afrikaans, English, or Zulu. Clients could respond in any language of their choice. Clients could request a callback at any time. The nurse could initiate calls at any time. A 2wT message sent or received using SMS was free. Routine data rates were applied for males who chose 2wT using WhatsApp. Automated messages, or “templates” sent through WhatsApp required approval by WhatsApp staff, and templates were approved separately for each language in which they were sent out.

2wT client protections were in place. 2wT participants could always seek in-person care when desired or for emergencies in line with South African National Department of Health VMMC guidelines. When there were power supply disruptions affecting cell connectivity, clients were not enrolled into 2wT and interested clients were followed up with standard of care through in-person visits. As with standard-of-care clients, if 2wT males did not respond within the first days (day 3 for males younger than 18 years and day 8 for males aged 18 years and older), a system alert “tasked” the nurse with calling the client. Clients who could not be reached by message or by phone were referred for routine tracing as per guidelines.

### Theoretical Framework

For the overall stepped wedge trial, we applied the implementation science framework, RE-AIM, to understand factors related to 2wT scale-up [28]. For this embedded process evaluation component, we explored 3 individual-level aspects related to the choice between SMS and WhatsApp: 2wT reach, effectiveness, and implementation. “Reach” was explored by looking at the proportion and characteristics of 2wT clients who chose WhatsApp versus SMS. “Effectiveness” was measured by comparing 2wT response rates by SMS and WhatsApp and understanding client preferences through quality improvement phone calls. We also explored an organizational-level aspect of the RE-AIM framework, “Implementation” summarizing feedback related to the technical considerations and constraints faced by the 2wT team using WhatsApp, informing future 2wT implementation improvements.

### Data Sources and Analysis

This paper reports on three data sources: (1) the 2wT database, (2) a quality improvement exercise in which study staff called a subset of 2wT participants to solicit feedback on the system, and (3) informal feedback from the 2wT technical team leading 2wT implementation.

#### Reach and Effectiveness: Quantitative Data Source and Analysis

The 2wT database included enrollment details including patient name, phone number, enrollment date, age, language preference, and messaging platform chosen (SMS or WhatsApp). The 2wT database also maintained records of daily responses, both inbound (from clients) and outbound (from the system or nurse). We analyzed 2wT response and interaction data using R Studio (version 2023.12.1+402; R Foundation for Statistical Computing). We assessed differences in message platform preference using a 1-sample *t* test. We included descriptive statistics about demographics, including counts and percentage of districts where the procedure was conducted, age groups and 2wT languages, and compared between platforms using chi-square tests. For response content, we presented a diagram of the frequencies of the response types by day. Responses included no AE, potential AE, direct interaction with the provider, and no response. When males responded with a “1” indicating they had a question or issues with healing, the provider followed up to ask for more details, and the following responses from males were captured as “direct interaction.”

For the response platform, we presented counts and percentages of those who responded within 3 days, because if there was no response by that time, the 2wT system prompted a health care provider to follow up. We also presented, “any response within 14 days,” as documentation of follow-up within 14 days is a President’s Emergency Plan for AIDS Relief (PEPFAR) Monitoring, Evaluation and Reporting (MER) indicator [34]. For days a response was requested, we presented counts and percentages and compared responses (including responses within 3 and 14 days) using a 2-sample proportion *z* test. Finally, we calculated the average number of responses for days a response was requested, by the platform, using a Welch 2-sample *t* test [35]. Both 95% CIs and df were reported.

#### Effectiveness: Client Satisfaction Through Quality Improvement Data Source and Analysis

For routine quality improvement data, the 2wT study team (a nurse and a study coordinator) called clients during this period, attempting to reach at least 100 males for feedback from among the most recent clients. The number 100 was chosen to reflect both a desire for broad feedback and scarce human resources. The team asked each participant 9 questions about their 2wT experience (interview guide attached in Multimedia Appendix 1) and recorded their responses in a Microsoft Excel spreadsheet. Responses were standardized and categorized by an external researcher. The team summarized responses quantitatively and categorized qualitative feedback by theme.

#### Implementation: WhatsApp Team Feedback Data Source and Analysis

Last, we collected information from a shared, web-based Google Document that contained team feedback during implementation. The team reviewed all feedback for WhatsApp-related experiences and summarized entries in tabular format.

### Ethical Considerations

The review boards of the University of Washington (number 00009703; CF) and the University of Witwatersrand, Human Research Ethics Committee (number 200204; GS) approved the study protocol for the routine scale-up of 2wT as part of the stepped wedge study. No specific consent was required for 2wT follow-up enrollment nor data collection as part of routine quality improvement activities. No client identification names or numbers were included in the analyzed data.

## Results

### Reach: Client Characteristics

**Figure 3:**
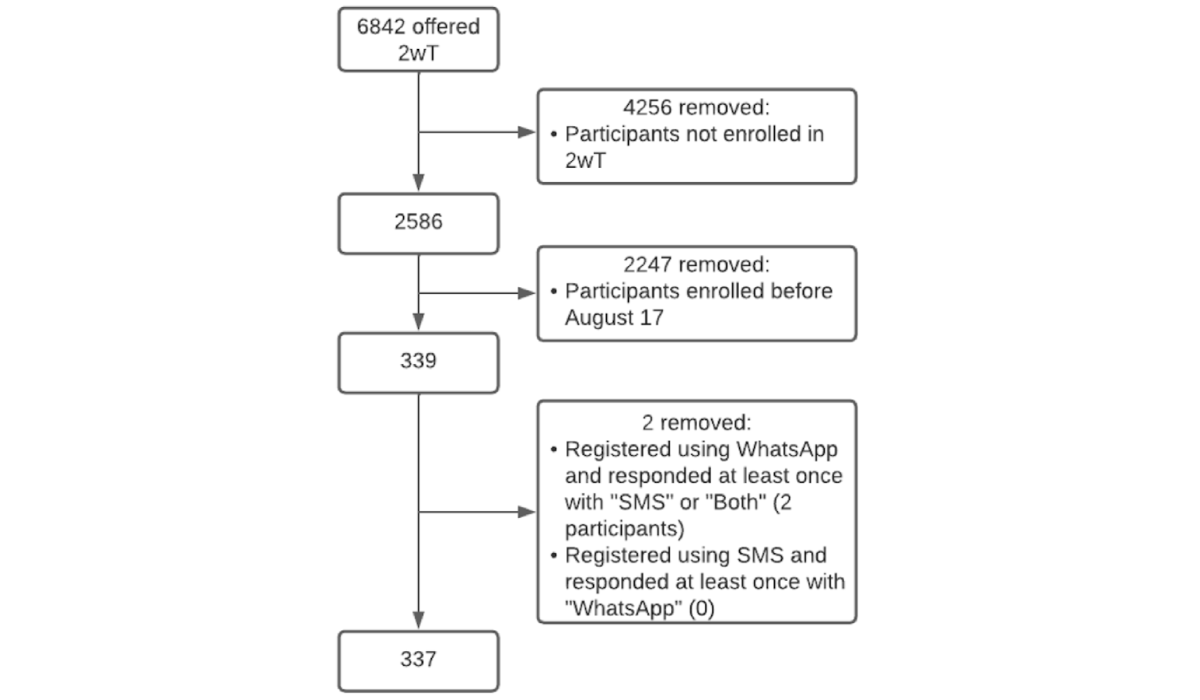
Exclusion diagram for routine and 2wT clients. This figure depicts the number of clients enrolled in 2wT and those included for analysis. In this overall study, 6842 clients were offered 2wT. Of 2586 participants who opted in, 339 were during the period where clients were offered a choice of WhatsApp or SMS. 2wT: 2-way texting.

Of the 6842 participants in the stepped wedge trial who were offered 2wT, 2586 (38%) participants opted in (Figure 3). Of all 2586 clients enrolled across the stepped wedge, 339 males enrolled from August 17 to October 19, 2023 (Table 1). Of the 339 males, 337 males were included in the process evaluation analysis after excluding 2 clients who enrolled in one platform and responded on the other.

There was no significant difference (1-sample *t* test, *t*_1_=0.76 *P*=.38) in the proportion who chose WhatsApp (177/337, 53%) versus SMS (160/337, 47%) (Table 1). Most clients were enrolled in Ekurhuleni District (297/337, 88%). The mean age was 30 (SD 10) years. Most males preferred English for their automated messages.

**Table 1:** Demographics of 2-way texting (2wT) participants during the period of platform choice.

		WhatsApp	SMS	Chi-square/ *t* test (*df*)	*P* value	Overall
**Province/District/ (Rural/Urban), n (%)**						
	Gauteng/Ekurhuleni/Urban	165 (93)	132 (83)	.57 (1)^a^	.45	297 (88)
	North West/Dr Kenneth Kaunda/Peri-urban or rural	12 (7)	28 (17)	4.17 (1)^a^	.04	40 (12)
Age (years), mean (SD)		29 (10)	31 (11)	N/A^b^	N/A^b^	30 (10)
**Age (years), n (%)**						
	15-29	96 (54)	76 (47)	2.33 (1)^a^	.13	172 (51)
	30+	81 (46)	84 (52)	.06 (1)^a^	.82	165 (49)
**Language category, n (%)^c^**						
	English	134 (76)	119 (74)	.89 (1)^a^	.35	253 (75)
	Setswana	N/A^d^	5 (3)	N/A^e^	N/A^e^	5 (1)
	Sesotho	N/A^d^	6 (4)	N/A^e^	N/A^e^	6 (2)
	Isizulu	43 (24)	30 (19)	2.32 (1)^a^	.13	73 (22)
Total		177 (53)	160 (47)	.76 (1)^f^	.38	337 (100)

^a^A chi-square test was conducted.

^b^Not applicable. A *t* test was not conducted because at least one group was not normally distributed.

^c^No clients selected Afrikaans.

^d^Not applicable. Setswana and Sesotho were not offered on WhatsApp.

^e^Not applicable. A chi-square test was not conducted due to small sample sizes.

^f^A 1-sample *t* test was conducted.

### Effectiveness

#### Responses

**Figure 4:**
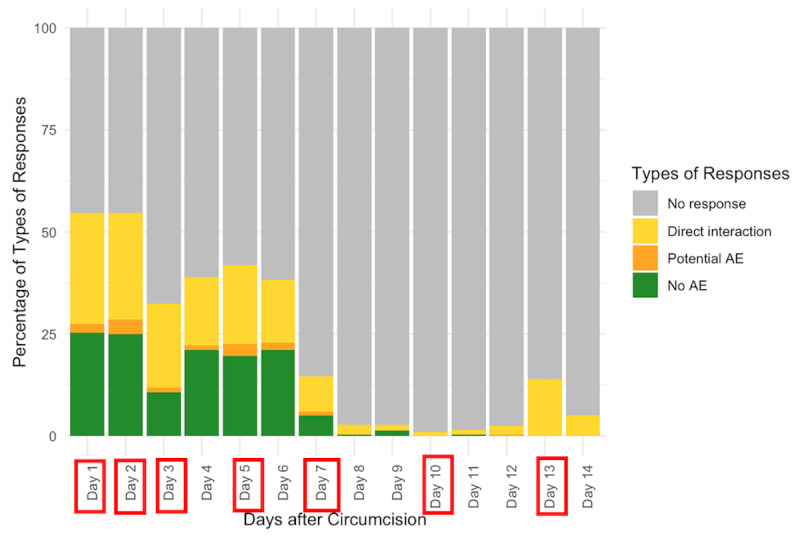
Distribution of types of responses to messages in the days after circumcision. Days with red boxes denote the days the 2-way texting system requested a response. These figures show the types of responses by males from days 1 to 14 after circumcision. The first is for all clients and the subsequent 2 are for clients registered by WhatsApp and SMS, respectively. On any given day, clients could respond in 1 of 4 ways: no response; no reported AE, reported potential AE or complication; or direct interaction with any text to the nurse. AE: adverse event.

**Figure 5:**
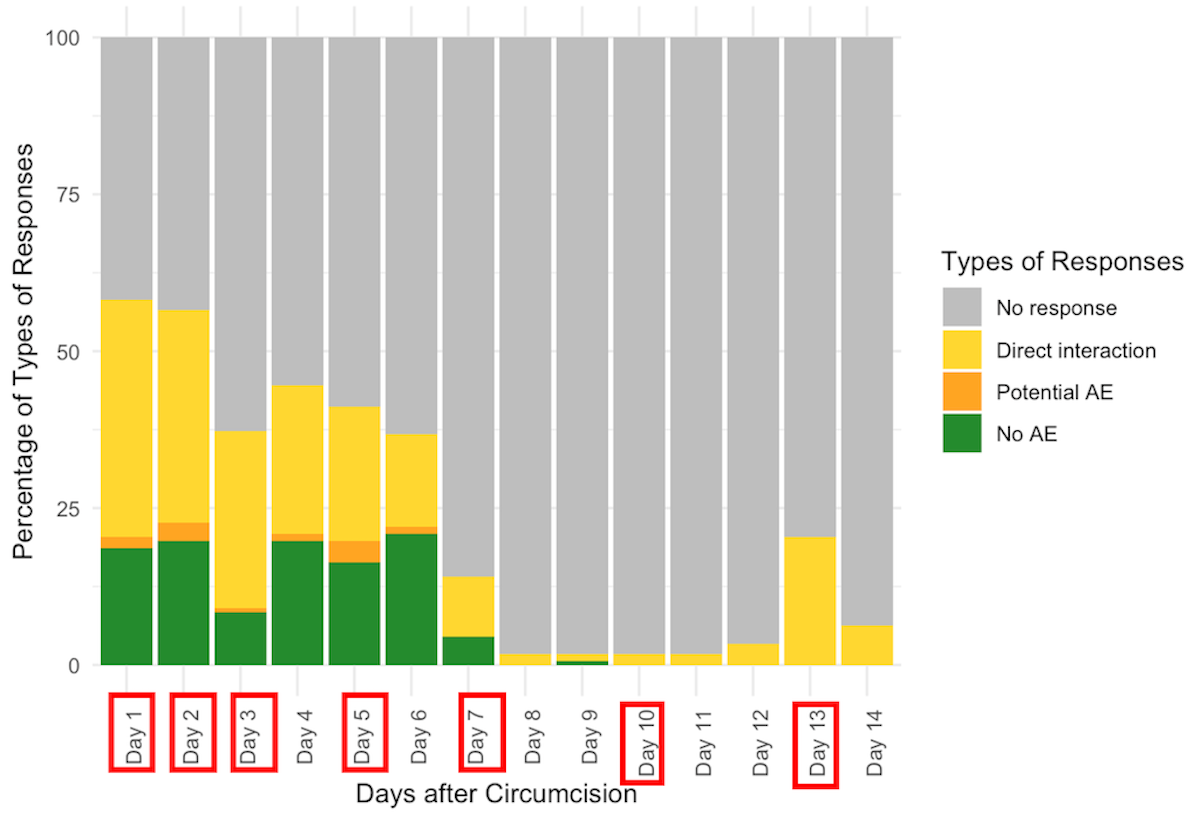
Daily response: WhatsApp clients. Days with red boxes denote the days the 2-way texting system requested a response. AE: adverse event.

**Figure 6:**
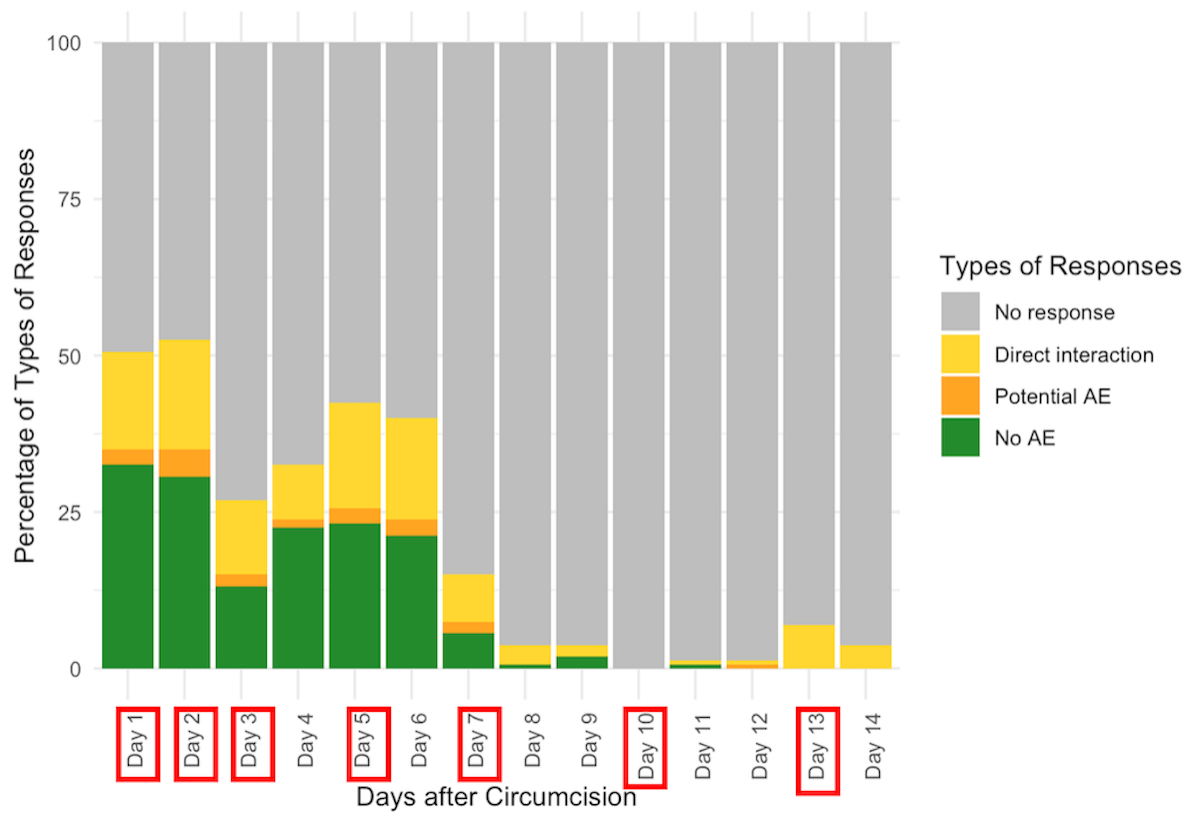
Daily response: SMS clients. Days with red boxes denote the days the 2-way texting system requested a response. AE: adverse event.

Overall, the average of the response rates for each of the 14 days was 22% (ranging from 1% on day 10 to 55% on days 1 and 2, Figure 4). Response rates did not vary significantly by 2wT delivery platform: WhatsApp clients responded to 23% of all messages (ranging from 2% on day 8 to 58% on day 1, Figure 5) and 20% of SMS clients (ranging from 0% on day 10 to 52% on day 2, Figure 6). On days when a system-generated message was sent requiring a response, the mean response rate was 30%.

On average, across all clients, 235 (70%) of males responded within 3 days (Table 2). This did not vary by delivery platform (*P*=.80). Among all clients, and in accordance with PEPFAR guidance for at least one follow-up contact within 14 days, 267 (79%) of all participants responded at least once over the course of the 14 days and this did not differ by platform (*P*=.73).

**Table 2:** Response rate to daily 2-way texting (2wT) prompts, by platform (WhatsApp or SMS).

Day of response requested (n=337)			WhatsApp		SMS	*z* test / *t* test (*df*)		*P* value		Overall
**Any response within X days, n (%)**										
	Any response ≤ 3 days	125 (71)		110 (69)		.07 (1)^a^	.80		235 (70)	
	Any response ≤ 14 days	142 (80)		125 (78)		.12 (1)^a^	.73		267 (79)	
**Response rate by day of expected response, n (%)**										
	1	103 (58)		81 (51)		1.65 (1)^a^	.20		184 (55)	
	2	100 (56)		84 (52)		.39 (1)^a^	.53		184 (55)	
	3	66 (37)		43 (27)		3.70 (1)^a^	.05		109 (32)	
	5	73 (41)		68 (42)		.02 (1)^a^	.90		141 (42)	
	7	25 (14)		24 (15)		.01 (1)^a^	.94		49 (15)	
	10	3 (2)		0 (0)		1.15 (1)^a^	.28		3 (1)	
	13	36 (20)		11 (7)		11.60 (1)^a^	<.001		47 (14)	
	Average response on days with response requested	58 (33)		44 (28)		.70 (11.86)^b^	.50		102 (30)	
Total clients			177		160	N/A^c^		N/A		337

^a^A 2-sample proportion *z* test was conducted.

^b^A Welch 2-sample *t* test was conducted.

^c^N/A: not applicable (a 1-sample *t* test was conducted and presented above in Table 1).

#### Client Satisfaction

Out of 363 males in the process evaluation, the team attempted to reach 188 males, of which 104 (55%) males responded to the phone call: five were excluded (two who noted they chose in-person review and three who did not know their platform). Out of the 99 participants, 48 (48%) participants chose WhatsApp, and 51 (52%) participants chose SMS (Table 3). No clients who chose WhatsApp explained their preference. Among those who chose SMS, the most common reasons for SMS preference were that they did not have WhatsApp, did not know about the WhatsApp option, SMS was free, their network access would not support WhatsApp, they perceived SMS to be easier to use, or they thought SMS was the same as WhatsApp.

Nearly all (87/89, 98%) were happy with their platform choice and 92 (99%) males would recommend 2wT to friends. Themes identified as common to 2wT, by either platform, were that 2wT made logistics easier (in avoiding trips to the clinic), helped with information on physical healing, provided a private way to communicate with clinicians, and clinicians provided emotional support. One client appreciated the educational value of the messages, “yes, at first [I] was scared but then the messages gave me clear steps.” Another said he would, “recommend texting because with a message I can read it more than once and follow it unlike a call.” 2wT also saved the men from having to go to the clinic, “… [it’s] easier to respond than going to clinic when you have no problem.”

**Table 3:** Quantitative summary of quality improvement calls from providers to clients.

Questions (n=99)			Chose WhatsApp, n (%)	Chose SMS, n (%)		Overall, n (%)
**1. How were you followed up after VMMC^a^? (n=99)**						
	WhatsApp	46 (96)		1 (2)	47 (48)	
	SMS	1 (2)		49 (96)	50 (51)	
	Did not receive messages	1 (2)		1 (2)	2 (2)	
**2. After circumcision, did you choose SMS or WhatsApp for your check-ups? (n=99)**						
	WhatsApp	48 (100)		N/A^b^	48 (48)	
	SMS	N/A^b^		51 (100)	5 (52)	
**3. Did you return to the clinic after circumcision? (n=98)**						
	No	36 (75)		46 (92)	82 (84)	
	Yes	12 (25)		4 (8)	16 (16)	
**4. Were you happy with your follow-ups? (n=89)**						
	No	1 (2)		1 (2)	2 (2)	
	Yes	46 (98)		41 (98)	87 (98)	
**5. Did you send an emoji? (n=42)**						
	No	42 (100)		N/A^c^	42 (100)	
	Yes	0 (0)		N/A^c^	0 (0)	
**6. Did you send a Voice note? (n=45)**						
	No	43 (96)		N/A^c^	43 (96)	
	Yes	2 (4)		N/A^c^	2 (4)	
**7. Did you send a photo? (n=45)**						
	No	45 (100)		N/A^c^	45 (100)	
	Yes	0 (0)		N/A^c^	0 (0)	
**8. Would you recommend this follow-up to your friends? (n=93)**						
	No	1 (2)		0 (0)	1 (1)	
	Yes	45 (98)		47 (100)	92 (99)	

^a^VMMC: voluntary medical male circumcision.

^b^Not applicable: these are mutually exclusive values.

^c^Not applicable: those who chose SMS could not send emojis, voice notes, or photos.

### Implementation

To explore considerations and constraints in 2wT implementation using WhatsApp, the Google sheet of daily notes, challenges, and solutions was summarized and categorized largely from the organization’s perspectives by the 2wT team to inform implementation improvement. Results are presented in Table 4.

**Table 4:** Summary of feedback from technical team on the use of WhatsApp for 2-way texting (2wT).

Challenge	Definition/explanation
Inconsistency in WhatsApp message template approvals	Templates are the combined automated message content and delivery schedule. WhatsApp approves all client message templates. There are no clear guidelines for how WhatsApp staff approve or reject the submitted messages. The WhatsApp team rejected about 40% of submitted templates, with some being approved in one language but rejected verbatim in another. Sometimes the team resubmitted the same messages to WhatsApp without changing the content, which garnered faster approvals, potentially as the review team was different.
Limitation on WhatsApp supported languages for automated messages	WhatsApp only supports 3 languages: Afrikaans, English, and Zulu. Five languages are supported by SMS. WhatsApp may exclude a significant number of clients from being supported via WhatsApp.
Complex integration of message aggregator and WhatsApp	Direct integration between RapidPro (the 2wT messaging service) and the WhatsApp Application Programming Interface is only available in versions 7.4 and after, requiring additional software to link the 2 applications for the complete intervention package. This introduced the extra cost of building, maintaining, testing, deploying, and monitoring an extra integration layer across the systems. Initially, a token was required to authorize the integration between RapidPro and WhatsApp once every 24 hours, creating a bottleneck to maintain 2wT implementation consistency.
WhatsApp is not designed for health care delivery	WhatsApp requires that business accounts open an advertising account within 30 days (even if there is no intention to advertise). This additional account is required to make changes to the WhatsApp templates.
Number of WhatsApp messages that can be sent is limited	WhatsApp requires a trial period, restricting initial message delivery frequency. The number of messages may increase over time.
No opt-out option for WhatsApp clients	The quality of templates is rated by clients using unclear parameters. Low ratings may be posted if clients report or block the 2wT messages. With low enough ratings, the template could be blocked or the 2wT intervention account disabled.

## Discussion

### Principal Findings

Through this process evaluation, we explored the use of WhatsApp versus SMS for 2wT-based, postoperative follow-up in routine VMMC settings. We found that 2wT clients were split in their preferences for either WhatsApp or SMS, but all clients appeared to like the 2wT telehealth approach. We found that 2wT response rates were also similar via SMS and WhatsApp, suggesting that either messaging platform facilitated engagement with clinicians. Feedback from the 2wT implementation team noted challenges using the WhatsApp platform as compared with SMS, constraints that may reduce the quality of 2wT implementation, and clinical oversight. The use of the RE-AIM framework helps interpret findings to provide guidance for improved reach, effectiveness, and implementation using WhatsApp at scale, informing other mHealth implementers in their consideration of SMS versus WhatsApp as a platform for telehealth delivery in routine LMIC settings.

For reach, platform preference was evenly split between WhatsApp and SMS, with similar demographics among those who chose each platform. We also found that males using either platform believed the 2wT intervention to be helpful and responded similarly, suggesting that both platforms may be viable for the 2wT approach. While these findings may simply reflect the proportion of males who own smartphones versus basic phones, it also indicates the need for equity in mHealth access. SMS-based 2wT reduces, rather than amplifies, inequities as WhatsApp is inaccessible to those who do not have a smartphone, money to buy data bundles, or access to 3G network [36]. Furthermore, SMS requires a low level of effort for engagement, while WhatsApp requires users to download the application and learn how to use it [37]. SMS may be more widely accessible to those who do not know how to use WhatsApp. WhatsApp should be considered as an additional platform choice alongside, but not replacing, SMS to maximize reach and equity as some males are only able to engage via SMS and others prefer WhatsApp.

For effectiveness, males responded similarly via WhatsApp and SMS with 70% (235/337) of participants responding within 3 days after VMMC and 79% (267/337) responding within 14 days after VMMC. For global VMMC donors and most national VMMC programs in SSA, critical follow-up dates after VMMC are 2 and 7 days after the procedure with required follow-up within 14 days [34] to ensure timely identification and management of AEs. While reported attendance at follow-up visits may be higher, previous VMMC studies have found between 60% and 80% of clients attended at least one visit [16,38,39]. Thus, follow-up via 2wT appears comparable to follow-up within routine care, with the added benefit of 2wT-based verification of those responses for monitoring and evaluation purposes.

For implementation, while clinicians and 2wT organizational level users found WhatsApp to be helpful, the application had several technical barriers that may reduce expansion potential. Due to challenges in setting up an aggregator for WhatsApp messaging, the technical team had to refresh a token in the system each day to ensure the messages flowed as intended, leading to some clients missing messages on days the technical team was unavailable. Furthermore, limited languages in WhatsApp, like unavailability of Sotho and Setswana, limit participation. Confirming message delivery via WhatsApp was more complex than for SMS-based messages, reducing fidelity assurances. Finally, WhatsApp clients who sent nontext messages (emojis and multimedia) did not have their responses registered in the 2wT system. If clients sent these types of responses, the response remained blank. This could have potentially caused HCWs to follow up with clients unnecessarily or resulted in clients incorrectly marked as nonresponders in the 2wT system.

In the future, several improvements should be considered. While 2wT allows for only text responses in either SMS or WhatsApp options, future 2wT adaptations could explore the use of emojis and voice notes, especially helpful for users who are less literate users. WhatsApp voice notes have proven helpful among providers [40] while emojis have been shown to be useful for assessing patients’ pain [41] suggesting that these areas merit future exploration. While WhatsApp allows users to send photos, due to privacy concerns and South Africa’s Protection of Personal Information Act [42], incorporation of photos into 2wT future is unlikely. Although no breeches in confidentiality were reported, vigilance in data security is always warranted. Last, as SMS-based clients can already send a message to “STOP” receiving messages, we hope that future WhatsApp advances will similarly support clients with an opt-out option to end participation if desired.

The findings of this exploration should be considered in light of several limitations. Some authors of this manuscript were part of the 2wT development team; although attention was paid to objectivity, including the involvement of external researchers, bias is possible. We report which platform males chose but cannot conclude that satisfaction was related to choice in platforms or the 2wT approach, itself. Deviations in fidelity could have altered 2wT implementation. In the future, we will examine fidelity as well as platform performance variations. Males who responded to the quality improvement calls had working phone numbers; those who were not reached could have responded differently. While the 2wT application itself is open-source (free without licensing costs), future costing analysis should consider setup and maintenance costs alongside running costs to better inform replication and expansion efforts. Last, this was a small-scale process evaluation with a small sample in 2 provinces of South Africa. Therefore, our results may not be generalizable to all settings.

### Conclusions

In this study, we use an implementation science approach to explore 2wT client preferences with WhatsApp or SMS, finding them to be near equal in terms of response rates and satisfaction. However, feedback from the 2wT implementation team suggests a few barriers to WhatsApp that may reduce enthusiasm for the messaging platform, including reduced language options and the need for clients to have a smartphone. We suggest that mHealth implementations for postoperative follow-up in SSA or other low-resource settings should use SMS as the primary messaging platform to increase equity, making WhatsApp available in addition to, and not instead of, SMS.

## Appendix

Multimedia Appendix 1 Quality improvement calls interview guide.

## Abbreviations

2wT: 2-way texting
AE: adverse event
HCW: health care worker
LMIC: low- or middle-income country
MER: Monitoring, Evaluation and Reporting
mHealth: mobile health
PEPFAR: US President’s Emergency Plan for AIDS Relief
RE-AIM: reach, effectiveness, adoption, implementation, and maintenance
SSA: sub-Saharan Africa
VMMC: voluntary medical male circumcision
WHO: World Health Organization

## Data Availability

The datasets generated and analyzed during this study, including demographics and SMS and WhatsApp text messaging response rates, are available from the corresponding author upon reasonable request.
