# Supplementary material for: WhatsApp Versus SMS for 2-Way, Text-Based Follow-Up After Voluntary Medical Male Circumcision in South Africa: Exploration of Messaging Platform Choice
Source: JMIR Form Res. 2024 Oct 16;8:e62762. doi: 10.2196/62762 (PMC11525085; doi:10.2196/62762)
Supplement: Multimedia Appendix 1 [file formative_v8i1e62762_app1.docx]

### Quality Improvement Calls Interview Guide

1. How were you followed up after VMMC?
2. After circumcision, did you choose SMS or WhatsApp for your check-ups?
3. Did you return to the clinic after circumcision?
4. Were you happy with your follow-ups?
5. Did you send an emoji?
6. Did you send a voice note?
7. Did you send a photo?
8. Would you recommend this follow-up to your friends?
9. How can we improve this texting system?
